# Supplementary material for: Dynamic Wetting Properties of Silica-Poly (Acrylic Acid) Superhydrophilic Coatings
Source: Polymers (Basel). 2023 Feb 28;15(5):1242. doi: 10.3390/polym15051242 (PMC10007625; doi:10.3390/polym15051242)
Supplement: Supplementary file 1 [file polymers-15-01242-s001.zip › polymers-2207815-supplementary.pdf]

Supporting Information for

Dynamic Wetting Properties of Silica-Poly (acrylic acid) Superhydrophilic Coatings

Sevil Turkoglu[1], Jinde Zhang[1], Hanna Dodiuk[2], Samuel Kenig[2], Jo Ann Ratto[3], Joey Mead[1*]

[1] Department of Plastics Engineering and Center for High-rate Nanomanufacturing, University of Massachusetts Lowell 40 University Avenue Lowell, MA 01854

[2] Department of Plastics Engineering, Shenkar College, Anne Frank St 12, Ramat Gan, İsrail

[3] US Army Combat Capabilities Development Command Soldier Center, Natick, MA 21005, USA

*Corresponding author, Joey_Mead@uml.edu

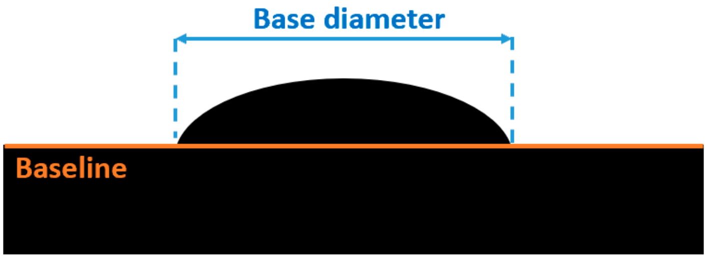

Figure S1. The sketch of a water droplet on a substrate

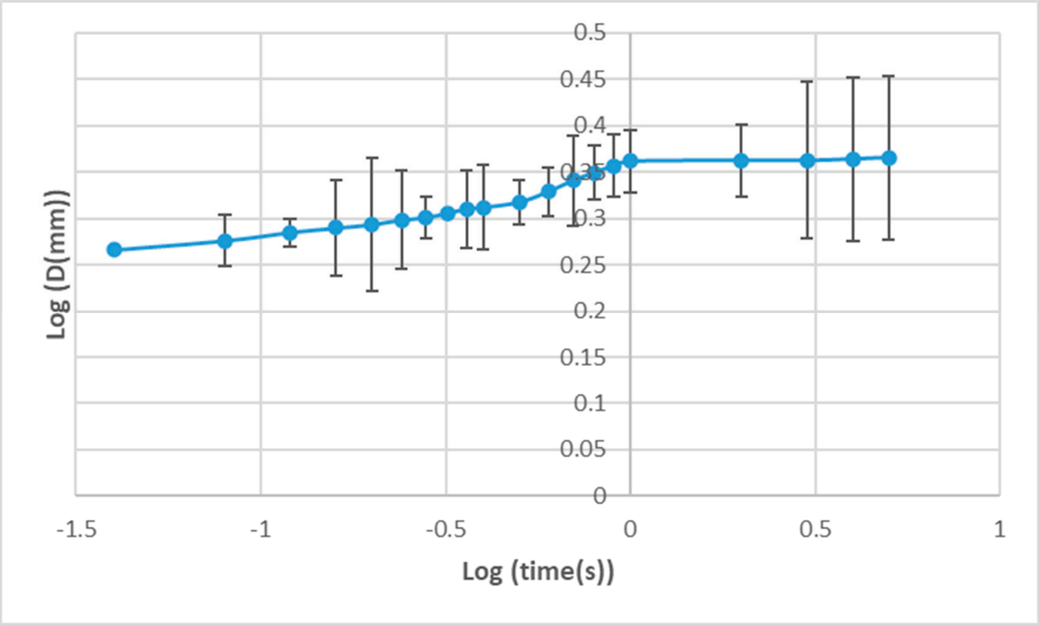

Figure S2. Plot of Log(D) vs Log(t) for PAA coating.

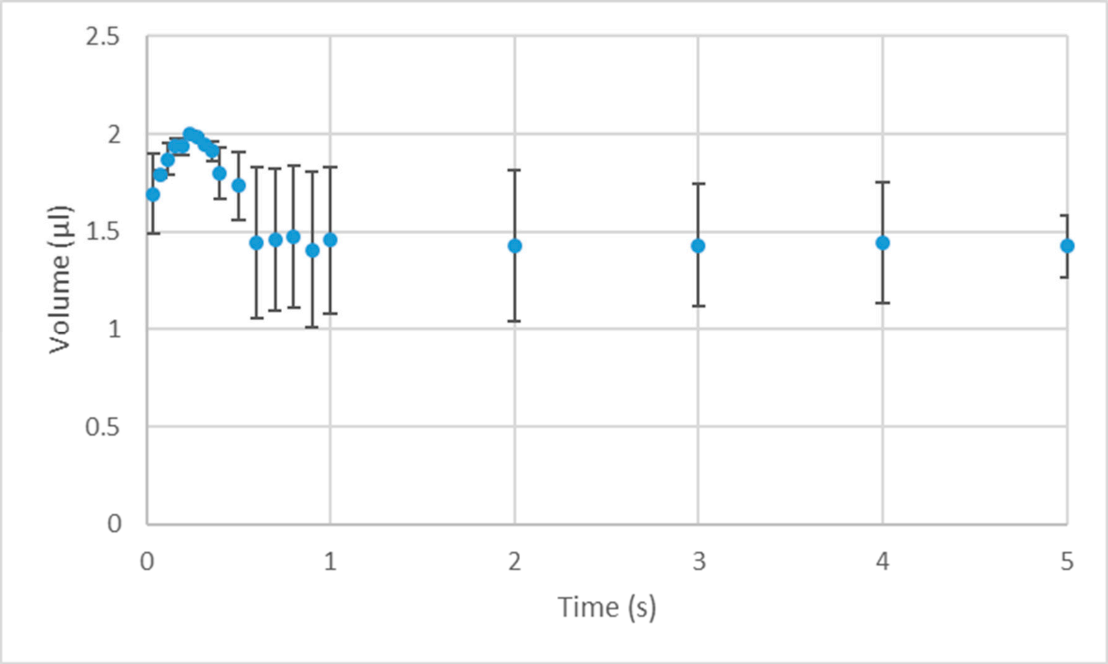

Figure S3. Plot of volume vs time for PAA coating.

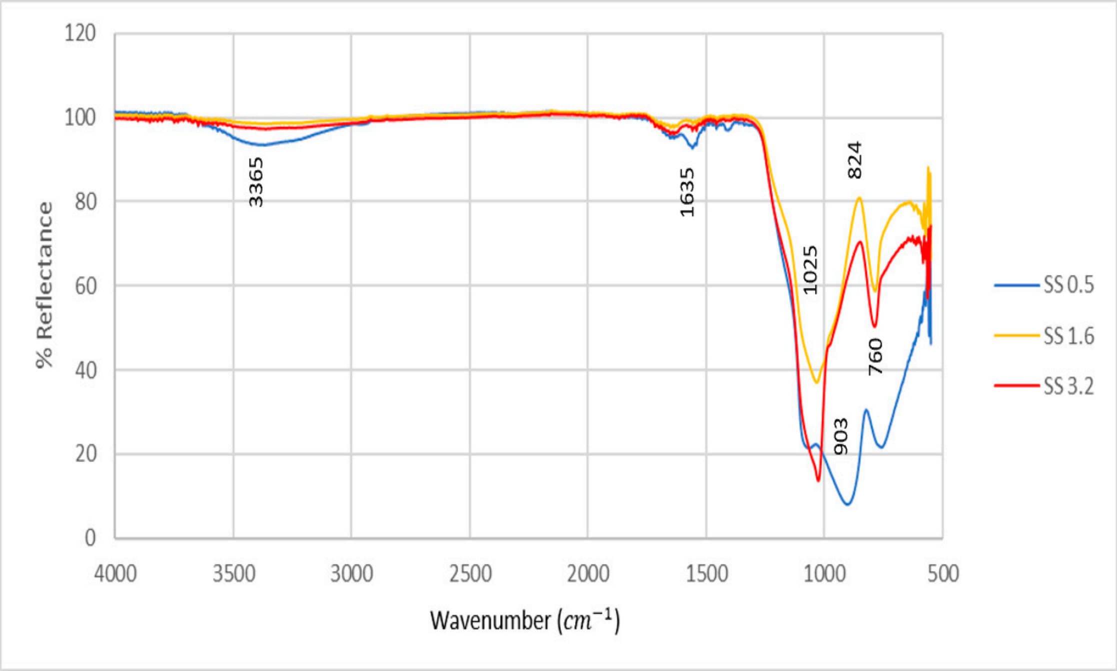

Figure S4. FTIR spectrum of several samples

Table S1. IR Absorptions and their assignments

**Wavenumber (cm^{-1})**	**Tentative assignment of functional group**
3365	OH stretching and hydrogen bonding
1025	Si–O–Si stretching
903	Si- OH
760	Si–O bending
1635	C=O stretching
1122	C-O stretching vibrations
824	C-H bending

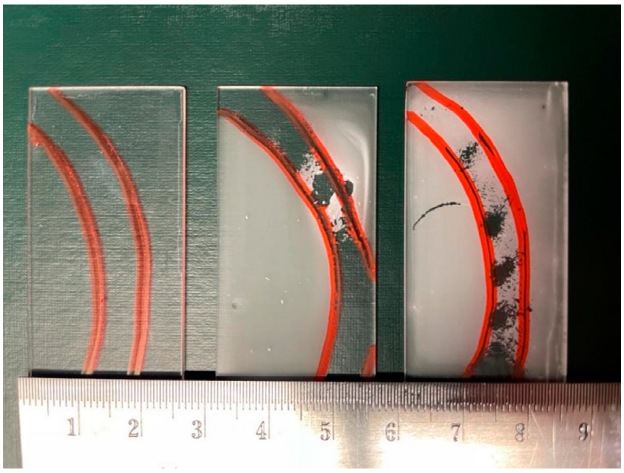

Figure S5. Top view images of the coatings after 30 cycles of taber test. Left (SS0.5), middle (SS1.6), right (SS3.2). The abraded area is demonstrated in between the orange lines.

Table S2. Work of adhesion values for the coatings after several abrasion cycles.

**Number of taber cycles**	**Work of adhesion (mN/m)**		
	**SS0.5**	**SS1.6**	**SS3.2**
**0**	145.42	145.50	145.42
**10**	137.67	137.09	139.31
**20**	134.55	132.45	135.86
**30**	130.18	130.18	130.18
